# Resuscitative endovascular balloon occlusion of the aorta for uncontrolled haemorrahgic shock as an adjunct to haemostatic procedures in the acute care setting

**DOI:** 10.1186/s13049-016-0205-8

**Published:** 2016-02-09

**Authors:** Junya Tsurukiri, Itsurou Akamine, Takao Sato, Masatsugu Sakurai, Eitaro Okumura, Mariko Moriya, Hiroshi Yamanaka, Shoichi Ohta

**Affiliations:** Emergency and Critical Care Medicine, Tokyo Medical University Hachioji Medical Center, 1163 Tatemachi, Hachioji, Tokyo 193-0998 Japan; Emergency and Disaster Medicine, Tokyo Medical University Hospital, 6-7-1 Nishi-shinjuku , Shinjuku, Tokyo 160-0023 Japan

**Keywords:** Emergency department, Shock, Intra-aortic balloon occlusion, Trauma, Gastrointestinal bleeding, Intensive care

## Abstract

**Background:**

Haemorrhagic shock is a major cause of death in the acute care setting. Since 2009, our emergency department has used intra-aortic balloon occlusion (IABO) catheters for resuscitative endovascular balloon occlusion of the aorta (REBOA).

**Methods:**

REBOA procedures were performed by one or two trained acute care physicians in the emergency room (ER) and intensive care unit (ICU). IABO catheters were positioned using ultrasonography. Collected data included clinical characteristics, haemorrhagic severity, blood cultures, metabolic values, blood transfusions, REBOA-related complications and mortality.

**Results:**

Subjects comprised 25 patients (trauma, *n* = 16; non-trauma, *n* = 9) with a median age of 69 years and a median shock index of 1.4. REBOA was achieved in 22 patients, but failed in three elderly trauma patients. Systolic blood pressure significantly increased after REBOA (107 vs. 71 mmHg, *p* < 0.01). Five trauma patients (20 %) died in ER, and mortality rates within 24 h and 60 days were 20 % and 12 %, respectively. No REBOA-related complications were encountered. The total occlusion time of REBOA was significantly lesser in survivors than that in non-survivors (52 vs. 97 min, *p* < 0.01). Significantly positive correlations were found between total occlusion time of REBOA and shock index (Spearman’s *r* = 0.6) and lactate concentration (Spearman’s *r* = 0.7) in survivors.

**Conclusion:**

REBOA can be performed in ER and ICU with a high degree of technical success. Furthermore, correlations between occlusion time and initial high lactate levels and shock index may be important because prolonged occlusion is associated with a poorer outcome.

## Background

Trauma and upper gastrointestinal bleeding (UGIB) are the most common causes of haemodynamic instability in patients with haemorrhage admitted to the emergency department (ED) and persistent haemorrhage is a major cause of death in acute care management [1–3]. Although the main aim of resuscitation is to stop the haemorrhage and restore circulating blood volume, persistent haemorrhage can be rapidly fatal. In such cases, conventional options for impending haemodynamic collapse are resuscitative thoracotomy (RT) and aortic clamping immediately performed in the emergency room (ER), particularly for uncontrolled torso haemorrhage or unstable pelvic fractures [4–8]. However, these procedures are invasive; therefore, resuscitative endovascular balloon occlusion of the aorta (REBOA) is increasingly used as an alternative to RT [9, 10]. The aim of REBOA is to maintain cerebral and coronary circulation to temporarily control arterial haemorrhage from the injured organ via occlusion using balloon inflation of the aortic lumen. Although a recent systematic review of REBOA in various clinical settings was found to successfully elevate central blood pressure in haemorrhagic shock, the effectiveness and indications for this intervention remain unclear [11]. Moreover, when applied as the only immediately available intervention for trauma patients with haemodynamic instability, REBOA was associated with increased mortality [12]. However, this study of trauma registry data was limited by the data elements collected in the registry, which mainly included age, vital signs and severity of injury. Therefore, we conducted a retrospective study of patients with haemorrhagic instability who underwent REBOA at a single emergency centre to determine the effect of REBOA on mortality and identify associations with vital indicators upon presentation at emergency facilities.

## Methods

### Patients and study design

The ethics committee of Tokyo Medical University Hachioji Medical Center approved the design of this retrospective study of patients with suspected haemorrhagic shock who subsequently underwent REBOA in ER or who were admitted to our intensive care unit (ICU) and subsequently developed haemorrhagic shock and underwent REBOA in ICU between September 2010 and September 2015. Patients with a systolic blood pressure (SBP) <90 mmHg or a shock index (SI; ratio of heart rate to SBP) ≥1.0 were considered to be in shock. We excluded patients aged <15 years and those who had cardiac arrest on admission or were diagnosed with any terminal disease during the study period.

### Intra-aortic balloon occlusion catheter

**Fig. 1:**
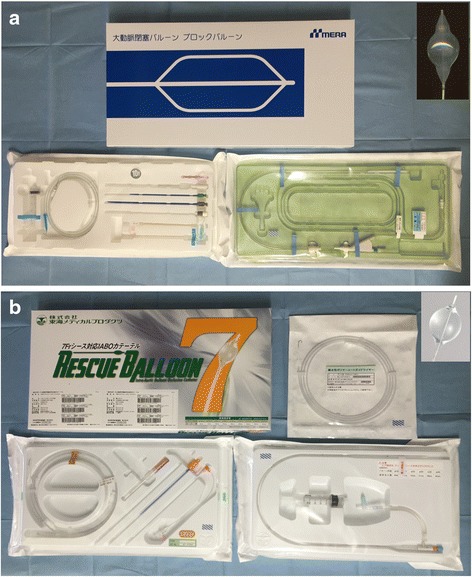
Intra-aortic balloon occlusion catheter available in Japan. **a** 10 Fr. BLOCK BALLOON™; **b** 7 Fr. RESCUE BALLOON®

In our ER, 10 Fr. intra-aortic balloon occlusion (IABO) catheters (BLOCK BALLOON™; Senko Medical Instrument, Tokyo, Japan) were used until 2014. Since 2014, 7 Fr. IABO catheters (RESCUE BALLOON®; Tokai Medical Products, Tokyo, Japan) have been available. For percutaneous deployment of IABO catheters, all necessary guidewires, sharps and introducers are packaged together in the kit (Fig. 1).

### Intervention

Treatment for haemorrhagic shock was performed on the basis of the Advanced Trauma Life Support resuscitation guidelines and the response to an initial fluid resuscitation with 1 L (since 9^th^ edition) or 2 L (until 8^th^ edition) of Ringer’s lactate. Acute care physicians categorised the patients into three categories based on subsequent haemodynamic responses to initial fluid resuscitation: a response group, showing immediate recovery from shock and remaining normotensive after initial fluid resuscitation; a transient-response group, showing recovery from shock, but an inability to remain normotensive after initial fluid resuscitation; and a non-response group, showing haemodynamic instability and no response to fluid resuscitation. Patients in the transient-response and non-response groups were considered to be haemodynamically unstable; therefore, empirical administration of blood and blood product transfusion were initiated earlier. Moreover, one or two acute care physicians performed all REBOA procedures. In our department, TJ was trained for ≥1 year as a member of the endovascular team in the Radiology Department of another university hospital, whereas all other acute care physicians in our ER performed sheath insertion using percutaneous femoral access >5 times under the guidance of TJ before performing REBOA.

For the REBOA procedure, a 10 Fr. (BLOCK BALLOON™) or 7 Fr. (RESCUE BALLOON®) sheath was first inserted into the femoral artery using the Seldinger method. In this study, all patients underwent sheath insertion via percutaneous femoral access. It was important that the sheath was passed over the wire into the femoral artery, and once the dilator and sheath had been advanced over the wire through the skin into the artery, the dilator was removed, leaving the sheath. When it was very difficult to pass the wire through either femoral artery, we discontinued the procedure and considered it to be a failed REBOA. After insertion of the femoral artery sheath, the IABO catheter was placed into the aorta and REBOA was performed. After insertion of the femoral artery sheath, an IABO catheter was inserted into the aorta, with selection of the aortic zone for occlusion according to the recommendations of Stannard et al. under ultrasonographic guidance [13]. Placement of the balloon is normally preformed in zone 1 (proximal part of the aorta, origin of the left subclavian artery to the celiac artery) in patients with suspected intra-abdominal haemorrhage, including UGIB. In patients with suspected haemorrhage from a confirmed pelvic fracture, the IABO catheters were placed in zone 3, (distal part of the aorta, lowest renal artery to the aortic bifurcation). IABO catheter positioning was performed under ultrasonographic guidance before REBOA placement and confirmed by portable chest or abdominal radiography in ER [14].

### Data collection

The following characteristics were noted from the charts and radiographs of all patients with haemodynamic instability: age, sex, vital signs, clinical history, haemorrhagic severity, blood cultures, metabolic values [pH, lactate concentration and base excess], blood transfusion, REBOA-related complications and mortality. In patients admitted to ER or ICU, blood cultures and metabolic values were measured at the beginning of resuscitative intervention.

### Statistical analyses

Data from all eligible patients were analysed. Continuous variables are shown as median values with interquartile ranges. Between-group differences were statistically assessed using the Mann − Whitney *U* test for continuous variables and the Fisher’s exact test for categorical variables. The Spearman correlation coefficient was used to identify correlations between the evaluated parameters. All statistical analyses were performed using Prism version 6.0a statistical software (GraphPad Software, San Diego, CA, USA). Categorical variables were calculated as the ratio (percentage) of the frequency of occurrence. A probability (*p*) value of > 0.05 was considered statistically significant.

## Results

### Demographics and clinical characteristics

Twenty-five patients (median age, 69 years; age range, 25 − 86 years; 62 % males), including 16 trauma and 9 non-trauma patients, were included in this study. The demographics and clinical characteristics of all patients are shown in Table 1. The aetiologies of the trauma and non-trauma patients are shown in Tables 2 and 3, respectively. The REBOA success rate was 90 % in this study. Five trauma patients (20 %) died in ER. The mortality rates within 24 h and 60 days were 20 % (trauma, *n* = 4; non-trauma, *n* = 1) and 12 % (trauma, *n* = 1; non-trauma, *n* = 2), respectively. The complications observed with REBOA were not encountered.

**Table 1:** Demographics and clinical characteristics of patients

Variables	Trauma (*n* = 16)	Non-trauma (*n* = 9)	Total (*n* = 25)
Age (y), median (IQR)	72 (39–82)	69 (63–72)	69 (45–80)
Male, n (%)	6 (38)	9 (100)^*^	15 (75)
Shock index, median (IQR)	1.4 (1.1–1.5)	1.6 (1.0–2.1)	1.4 (1.1–1.16)
Injury severity score, median (IQR)	41 (33–49)	-	-
Glasgow-Blatchford score, median (IQR)	-	-	-
Systolic blood pressure before REBOA (mmHg), median (IQR)	78 (67–87)	64 (61–77)	71 (62–87)
Base excess (mmol/L), median (IQR)	-9.0 (-18.7–-6.3)	-11.5 (-14.6–-9.2)	-9.4 (-15.1–-6.4)
pH, median (IQR)	7.33 (7.25–7.41)	7.30 (7.23–7.38)	7.32 (7.23–7.39)
Lactate (mg/dL), median (IQR)	4.3 (3.2–9.0)	6.3 (5.6–11.0)	5.7 (3.7–11.0)
Prothrombin time (%), median (IQR)	64.5 (46.5–79.5)	67.0 (51.0–7.30)	67.0 (48.0–77.0)
Activated partial thromboplastin time (sec), median (IQR)	56.3 (41.4–75.9)	39.3 (35.3–64.5)	53.4 (38.2–75.7)
Insertion at the ER, n (%)	16 (100)	6 (67)	22 (88)
Failed REBOA, n (%)	3 (19)	0	3 (12)
Total occlusion time of REBOA (min), median (IQR)	65 (57–99)	55 (50–95)	61 (51–98)
PRBC transfusion within 24 h (mL), median (IQR)	1540 (840–2590)	1960 (1400–2800)	-
FFP transfusion within 24 h (mL), median (IQR)	720 (360–1440)	900 (720–1440)	-
Outcomes, n (%)			
Died at the ER	5 (31)	0	5 (20)
Died within 24 h	4 (25)	1 (11)	5 (20)
Died within 2 months	1 (6)	2 (22)	3 (12)

*IQR* interquartile range, *APACHE* acute physiology and chronic health evaluation, *ER* emergency room, *ICU* intensive care unit and *REBOA* resuscitative endovascular balloon occlusion of the aorta; ^*^ *p* < 0.05 vs. trauma group

**Table 2:** Characteristics of trauma patients

No.	Age (y)	Sex	Mechanism	SI	ISS	Injury (AIS > 3)						Sheath insertion	Position (Zone)	Intervals for REBOA (min)	REBOA-related complications	Outcome			Cause of death
						Head	Chest	Abdomen	Pelvis	Vertebral	Extremity					ER	>24 h	>2 months	
1	34	F	Fall	1.5	29	-	+	-	Stable	-	+	Success	III	65	None	Alive	Alive	Alive	-
2	45	M	Laceration	1.5	9	-	-	-	-	-	Popliteal artery and vein tear	Success	III	70	None	Alive	Alive	Alive	-
3	21	M	TA (motorcycle)	1.1	16	-	-	Spleen: Grade IV	-	-	-	Success	II	40	None	Alive	Alive	Alive	-
4	78	F	TA (pedestrian)	1.4	41	+	+	-	Unstable	-	-	Success	III	43	None	Alive	Alive	Alive	
5	19	F	TA (motorcycle)	1.6	50	-	+	Liver: Grade III	-	-	+	Success	I	62	None	Alive	Alive	Alive	-
6	52	M	Fall	0.9	41	+	+	Liver: Grade IV	-	+	-	Success	I	48	None	Alive	Alive	Alive	-
7	71	F	TA (pedestrian)	1.0	57	+	+	Spleen: Grade IV	Unstable	-	-	Success	I	99	None	Dead	-	-	Exsanguination
8	85	M	TA (motorcycle)	1.4	41	-	+	-	Unstable	-	-	Success	III	60	None	Dead	-	-	Exsanguination
9	73	F	Fall	1.9	57	+	+	-	Unstable	+	-	Success	II	65	None	Dead	-	-	Exsanguination
10	86	F	TA (pedestrian)	1.1	48	+	+	-	Unstable	-	-	Fail	II	-	None	Dead	-	-	Exsanguination
11	82	F	TA (pedestrian)	1.1	50	+	-	Spleen: Grade III	Unstable	-	+	Fail	II	-	None	Dead	-	-	Brain dead
12	76	F	TA (pedestrian)	1.2	25	-	-	Kidney: Grade IV	-	-	+	Fail	I	-	None	Alive	Dead	-	Exsanguination
13	41	F	Fall	2.2	34	-	+	Spleen: Grade III	Stable	-	-	Success	I	135	None	Alive	Dead	-	Exsanguination
14	83	F	Fall	1.5	34	+	Pulmonary vein injury	-	-	+	-	Success	I	124	None	Alive	Dead	-	ARDS
15	84	M	TA (pedestrian)	1.2	41	+	-	Spleen: Grade IV	-	-	-	Success	II	57	None	Alive	Dead	-	Brain dead
16	24	M	TA (motorcycle)	1.3	41	+	Brachial tear	-	-	-	-	Success	I	110	None	Alive	Alive	Dead	Brain dead

*SI* shock index, *ISS* injury severity score, *AIS* abbreviated Injury Scale, *REBOA* resuscitative endovascular balloon occlusion for the aorta, *ER* emergency room, *TA* traffic accident and *ARDS* acute respiratory distress syndrome

**Table 3:** Characteristics of non-trauma patients

No.	Age	Sex	SI	Glasgow-Blatchford score	Clinical Rockall score	Diagnosis	Treatment	Sheath insertion	Position (Zone)	Sheath insertion	CPA during procedure	Intervals for REBOA (min)	REBOA-related complications	Outcome			Cause of death
														ER	24 h>	3 months>	
17	68	M	1.6	13	3	Gastric ulcer	Surgery	Success	I	Success	No	46	None	Alive	Alive	Alive	-
18	50	M	1.0	12	2	Gastric ulcer	AE (failed endoscopy)	Success	I	Success	No	50	None	Alive	Alive	Alive	-
19	63	M	2.1	11	3	Pseudo-aneurysm by pancreatic fistula	AE	Success	I	Success	Yes	54	None	Alive	Alive	Alive	-
20	83	M	2.1	19	4	Duodenal ulcer	Endoscopy	Success	I	Success	No	140	None	Alive	Alive	Alive	-
21	36	M	0.7	7	3	Gastric ulcer	Endoscopy	Success	I	Success	No	20	None	Alive	Alive	Alive	-
22	69	M	2.8	17	3	Gastric ulcer	Endoscopy	Success	I	Success	No	57	None	Alive	Alive	Alive	-
23	72	M	1.2	9	3	Gastric ulcer/ Cerebral infarction	AE (failed endoscopy)	Success	I	Success	Yes	55	None	Alive	Alive	Dead	Exsanguination
24	69	M	1.7	12	3	Duodenal ulcer	AE (failed endoscopy)	Success	I	Success	Yes	95	None	Alive	Alive	Dead	Ischemic encephalopathy
25	80	M	0.8	14	5	Duodenal ulcer	AE (failed endoscopy)	Success	I	Success	No	145	None	Alive	Dead	-	Exsanguination

*SI* shock index, *CPA* cardiopulmonary arrest, *REBOA* resuscitative endovascular balloon occlusion of the aorta, *ER* emergency room; and *AE* angioembolizatoin

With regard to clinical characteristics, there were no significant differences in pH, lactate concentration, base excess, prothrombin time, activated partial thrombin time or injury severity score (ISS) between the survivors (*n* = 12) and non-survivors (*n* = 13). However, in trauma patients, there were significant differences in the revised trauma scale (RTS) and the trauma and injury severity scores (TRISS) between survivors and non-survivors (Table 4).

**Table 4:** Comparison of survivors and non-survivors

Variables	Survivors	Non-survivors	Total (*n* = 25)
Trauma, n (%)	6 (38)	10 (63)	-
Non-trauma, n (%)	6 (67)	3 (33)	-
Injury severity score, median (IQR)	35 (19–41)	41 (36–50)	-
Trauma and injury severity score, median (IQR)	0.79 (0.46–0.94)^*^	0.23 (0.15–0.44)	-
Revised trauma score, median (IQR)	5.56 (5.17–6.43)^*^	6.13 (4.03–6.38)	-
SBP before REBOA (mmHg), median (IQR)			
Trauma	87 (85–89)	67 (60–74)	78 (67–87)
Non-trauma	63 (60–84)	67 (65–72)	64 (61–77)
Total	86 (63–89)	67 (61–75)	71 (62–87)
SBP after REBOA (mmHg), median (IQR)			
Trauma	115 (106–123)	78 (72–100)	104 (78–118)
Non-trauma	104 (93–123)	112 (106–137)	111 (97–127)
Total	112 (101–126)^**^	95 (74–111)^**^	107 (90–118)^**^
ΔSBP (mmHg), median (IQR)			
Trauma	26 (19–46)	11 (8–30)	22 (11–32)
Non-trauma	35 (28–37)	49 (41–67)	37 (32–49)
Total	31 (22–41)	30 (11–45)	31 (19–46)
Blood transfusion (mL), median (IQR)			
Trauma	840 (840–2310)	1960 (980–2450)	1540 (840–2590)
Non-trauma	2240 (1540–2730)	1960 (1260–2380)	1960 (1400–2800)
Total	1680 (840–2800)	1960 (840–2520)	1960 (840–2800)
Occlusion time of IABO catheter (min), median (IQR)			
Trauma	55 (44–64)	99 (63–117)	65 (57–99)
Non-trauma	54 (47–56)	95 (75–120)	55 (50–95)
Total	52 (45–63)^*^	97 (61–121)	61 (51–98)

*IQR* interquartile range, *SBP* systolic blood pressure, *REBOA* resuscitative endovascular balloon occlusion of the aorta, and *IABO* intra-aortic balloon occlusion; ^*^ *p* < 0.05 vs. non-survivors; ^**^ *p* < 0.01 vs. SBP before REBOA in each group

### Changes in acute care management with REBOA

Table 4 shows that SBP was significantly higher after initiation of REBOA. However, there were no significant differences in ΔSBP (SBP after REBOA − SBP before REBOA) or the volume of blood transfusion between the survivors and non-survivors.

**Fig. 2:**
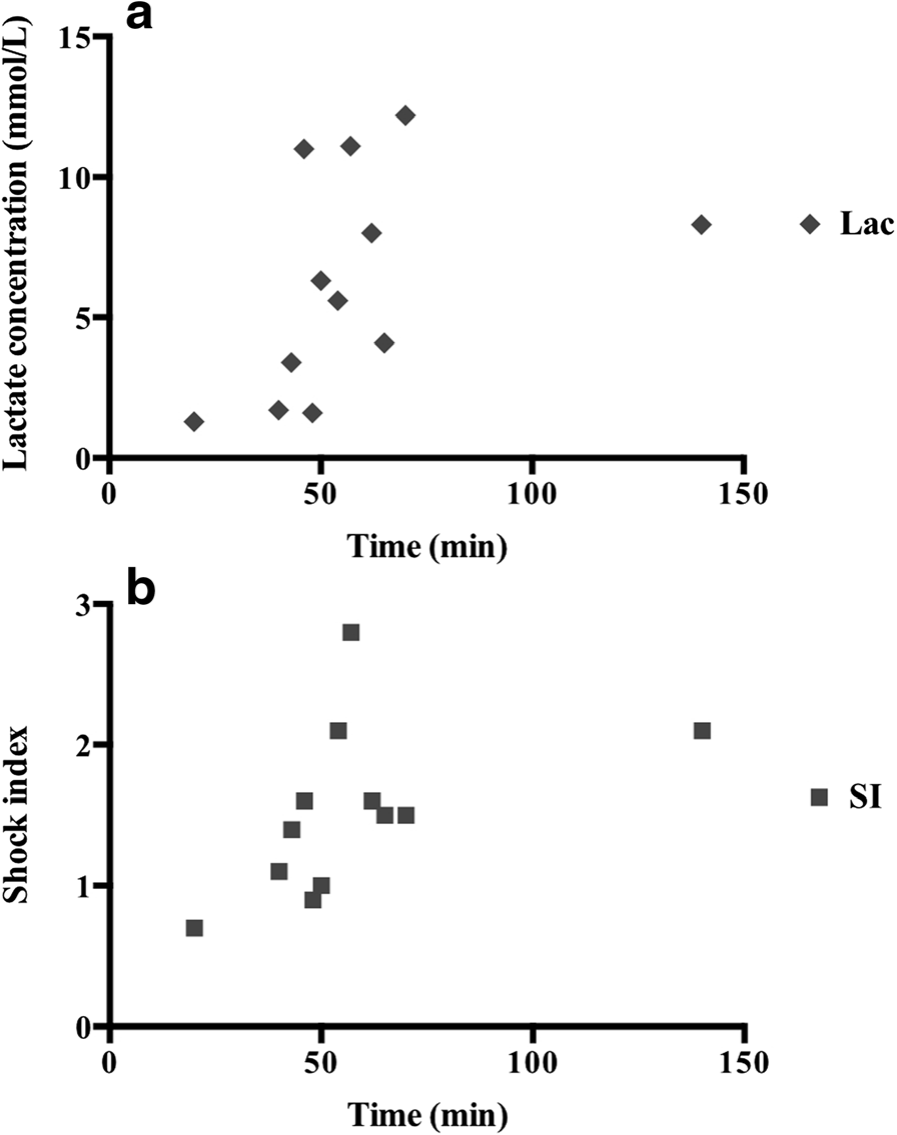
Correlation between the total occlusion time of the intra-aortic balloon occlusion catheter and lactate concentration/shock index. **a** Lactate concentration; **b** shock index

The total occlusion time of IABO catheters for the survivors was significantly shorter than for the non-survivors. Furthermore, strong positive correlations were found between total occlusion time and lactate concentration (Spearman’s *r* = 0.7, *p* = 0.02) and between total occlusion time and SI (Spearman’s *r* = 0.6, *p* = 0.04) in survivors (Fig. 2). We did not determine correlations between the occlusion time and any clinical variable in non-survivors.

### Analysis in cases of failed REBOA

**Fig. 3:**
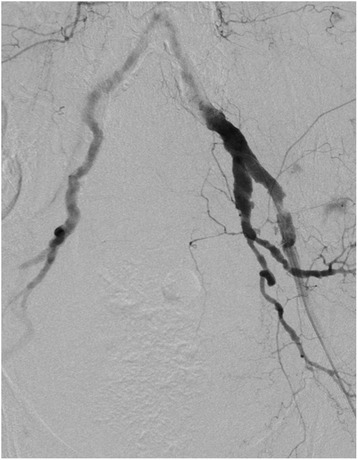
Angiography of an elderly trauma patient with failed REBOA revealed severe tortuosity of the femoral arteries

The REBOA procedure failed in three patients (patients 10–12 in Table 2) because the wire could not be blindly passed through the femoral artery for sheath insertion in ER. Two of the three patients underwent RT in ER and one underwent REBOA in the angiography suite. All three patients were aged >75 years with severe visceral injuries and pelvic fractures; therefore, they underwent angio-embolization for the pelvic injuries and angiography revealed severe tortuosity or torsion of the femoral arteries (Fig. 3).

## Discussion

In the present study, we found that the total occlusion time of REBOA was longer in non-survivors than that in survivors with similar results between trauma and non-trauma patients. For trauma patients, our results were consistent with those reported by Saito et al. and Irahara et al. [7, 8]. Furthermore, the median REBOA occlusion time of approximately 60 min was similar between trauma and non-trauma patients. A recent study reported a REBOA duration of >90 min in an animal model of haemorrhage-induced organ dysfunction, particularly that of the kidneys and liver [15]. However, REBOA for 60 min was reportedly well tolerated in an animal model of persistent haemorrhagic shock [16]. Although it is important to shorten the occlusion time of REBOA as much as possible, our results strongly support those observed in these previous animal studies.

Norii et al. reported increased mortality using REBOA in trauma patients with a significantly higher ISS and lower RTS compared with patients who did not undergo REBOA treatment, thus REBOA is the only immediately available option for trauma patients with haemodynamic instability in Japan [12]. The Japanese trauma registry data were limited (i.e., age, vital signs, prehospital records, and severity of injury) and several details of the clinical parameters, such as blood cultures, lactate levels, the volume of fluid transfusion, and clinical occlusion time of REBOA, were not provided. We found significant correlations between the total occlusion time of REBOA and lactate concentration and SI measured at the beginning of resuscitation in survivors. Lactate concentration and SI are more effective indicators than vital signs, such as heart rate and SBP, to predict mortality of trauma patients [17, 18]. DeMuro et al. advocated an SI >0.8 to identify trauma patients that will require intervention for haemostasis [19]. For UGIB, a high SI is associated with fatal haemorrhage [20]. These parameters are useful to confirm suspected massive hemorrhage and may be helpful for future consideration for introducing REBOA. In the present study, three cases with a high SI experienced cardiac arrest before IABO catheter insertion and two died (multiple organ failure because of exsanguination and ischemic brain injury, respectively). We speculate that the early introduction of an IABO catheter without inflation for preventive reasons is warranted in patients with haemodynamic instability and a high SI and/or lactate concentration. The Japanese Society of Diagnostic and Interventional Radiology in Emergency Critical Care and Trauma (DIRECT) is currently enrolling patients in a prospective multi-institutional trail (UMIN000015722) to examine the utility of and identify indications for REBOA.

A systematic review by Morrison et al. reported that REBOA successfully elevated central blood pressure in haemorrhagic shock in various clinical settings with [11]. Acute care physicians encounter haemorrhagic shock because of various causes, including ruptured abdominal aortic aneurysms, spontaneous bleeding caused by anticoagulation, and postpartum bleeding secondary to placenta previa or placenta abruption. Although endoscopic treatment for UGIB is generally acceptable, it can be difficult to completely achieve in some patients, and persistent haemorrhage can result in rapid death. A recent study demonstrated that advanced age, haemorrhagic shock, in-hospital bleeding, re-bleeding, and the need for surgery or other interventions are predictors of in-hospital mortality among patients with UGIB [21]. Moreover, patients with haemorrhagic shock on admission have an approximately 5-fold greater risk of intractability after initial endoscopic treatment, although mortality rates associated with UGIB have remained essentially unchanged at 5 % − 8 % [22]. Our results indicate the clinical safety and feasibility of REBOA in non-trauma patients, which have been insufficiently described in previous reports [23].

The complications observed with REBOA were caused by the insertion of the IABO catheter and femoral artery sheath. The major complications of IABO catheter insertion are vessel injuries (i.e., aortic dissection, rapture and perforation), embolisation, air emboli and peripheral ischaemia. However, Brenner et al. and Ogura et al. have reported no vessel injuries caused by an IABO catheter or inflated balloon in trauma patients [4, 7]. In the present study, there was no complication caused by the IABO catheter itself. In our department, we routinely use ultrasonography to guide positioning of the IABO catheter during procedures and evaluate catheter placement using portable radiography after catheter deployment. The findings of Guliani et al. support this result in their study showing that ultrasonography alone is as safe and accurate as fluoroscopy for positioning and deployment of an IABO catheter [14]. The major complications of sheath insertion are femoral artery injuries (i.e., pseudo-aneurysm, arteriovenous fistula and dissection) and lower limb ischaemia at the same site. Saito et al. reported a severe complication of lower limb ischaemia at the puncture site [7]. Therefore, we recommend careful consideration of ischaemic complications, particularly limb or organ ischaemia, during sheath insertion.

In the present study, we encountered difficulties with the insertions of a 10 Fr. sheath in three elderly trauma patients who were all women aged >75 years with severe tortuosity or torsion of the femoral arteries. Recent studies confirmed that the female sex and age >75 years are risk factors for femoral artery complications after endovascular treatment [24]. Other statistically significant risk factors included high body mass index, low platelet count, urgent procedures, increasing sheath size and administration of antithrombotic agents [25]. In such cases, performing endovascular procedures can be time-consuming and may be an issue with regard to the time required for resuscitation. In the acute care setting, the REBOA procedure is usually conducted blindly by acute care physicians, including emergency physicians and trauma surgeons, who must have sufficient knowledge of the risk factors and procedures associated with an endovascular approach. The development of new devices that do not require an oversized sheath, such as 10 Fr., or long guidewires is likely to reduce not only complications but also time to occlusion. In Japan, 7 Fr. IABO catheters have been clinically available since 2014, and we have used this device with technical success (100 %) by trained acute care physicians.

This study had several limitations, particularly the small number of evaluated patients sustaining different types of haemorrhagic shock, such as single trauma, multiple trauma and non-trauma (UGIB alone). Second, this was not a randomised, controlled trial or retrospective study using propensity scores because in the acute care setting, it is difficult to perform a randomised trial. Furthermore, use of a propensity score may be not suitable for a small sample size (trauma, *n* = 16 and non-trauma, *n* = 9) in a single emergency centre [26, 27]. Third, >30 % of the trauma patients in this study were aged ≥65 years and the clinical characteristics of such patients in Japan may differ from those in other countries [28]. Bernard et al. recently reported a population based analysis using Trauma Audit and Research Network data and the median age of trauma patients in whom may be potentially utilized REBOA was 43 years [29]. Thus, population based studies are beneficial to further evaluate the utility of REBOA for trauma populations.

## Conclusion

In our experience, REBOA can be performed in ER and ICU and achieve a high degree of technical success. Furthermore, the correlations of occlusion time and initial high lactate levels and SI may be important because prolonged occlusion is associated with a poorer outcome. Further large clinical studies to identify patient subgroups with haemodynamic instability because of traumatic and non-traumatic haemorrhage that may benefit from REBOA are warranted.

## Abbreviations

ED: emergency department
ER: emergency room
IABO: intra-aortic balloon occlusion
ICU: intensive care unit
ISS: injury severity score
REBOA: resuscitative endovascular balloon occlusion for the aorta
RT: resuscitative thoracotomy
RTS: revised trauma score
SBP: systolic blood pressure
SI: shock index
TRISS: trauma and injury severity score
UGIB: upper gastrointestinal bleeding
