# Erratum to: Resuscitative endovascular balloon occlusion of the aorta for uncontrolled haemorrahgic shock as an adjunct to haemostatic procedures in the acute care setting

**DOI:** 10.1186/s13049-016-0264-x

**Published:** 2016-05-16

**Authors:** Junya Tsurukiri, Itsurou Akamine, Takao Sato, Masatsugu Sakurai, Eitaro Okumura, Mariko Moriya, Hiroshi Yamanaka, Shoichi Ohta

**Affiliations:** Emergency and Critical Care Medicine, Tokyo Medical University Hachioji Medical Center, 1163 Tatemachi, Hachioji, Tokyo, 193-0998 Japan; Emergency and Disaster Medicine, Tokyo Medical University Hospital, 6-7-1 Nishi-shinjuku, Shinjuku, Tokyo, 160-0023 Japan

## Erratum

After publication of the original article [1], it came to the authors’ attention that there were some errors affecting Tables 1 and 3. These errors do not affect the scientific conclusion of the study presented in the original article.

**Table 1:** Demographics and clinical characteristics of patients

Variables	Trauma (*n* = 16)	Non-trauma (*n* = 9)	Total (*n* = 25)
Age (y), median (IQR)	72 (39–82)	69 (63–72)	69 (45–80)
Male, n (%)	6 (38)	9 (100)*	15 (75)
Shock index, median (IQR)	1.4 (1.1 − 1.5)	1.6 (1.0 - 2.1)	1.4 (1.1 − 1.6)
Injury severity score, median (IQR)	41 (33–49)	−	−
Glasgow-Blatchford score, median (IQR)	−	12 (11–14)	−
Systolic blood pressure before REBOA (mmHg), median (IQR)	78 (67–87)	64 (61–77)	71 (62–87)
Base excess (mmol/L), median (IQR)	−9.0 (−18.7 − -6.3)	−11.5 (−14.6 − -9.2)	−9.4 (−15.1 − -6.4)
pH, median (IQR)	7.33 (7.25 − 7.41)	7.30 (7.23 − 7.38)	7.32 (7.23 − 7.39)
Lactate (mg/dL), median (IQR)	4.3 (3.2 − 9.0)	6.3 (5.6 − 11.0)	5.7 (3.7 − 11.0)
Prothrombin time (%), median (IQR)	64.5 (46.5 − 79.5)	67.0 (51.0 − 73.0)	67.0 (48.0 − 77.0)
Activated partial thromboplastin time (sec), median (IQR)	56.3 (41.4 − 75.9)	39.3 (35.3 − 64.5)	53.4 (38.2 − 75.7)
Insertion at the ER, n (%)	16 (100)	6 (67)	22 (88)
Failed REBOA, n (%)	3 (19)	0	3 (12)
Total occlusion time of REBOA (min), median (IQR)	65 (57–99)	55 (50–95)	61 (51–98)
PRBC transfusion within 24 h (mL), median (IQR)	1540 (840–2590)	1960 (1400–2800)	−
FFP transfusion within 24 h (mL), median (IQR) Outcomes, n (%)	720 (360–1440)	900 (720–1440)	^−^
Died at the ER	5 (31)	0	5 (20)
Died within 24 h	4 (25)	1 (11)	5 (20)
Died within 2 months	1 (6)	2 (22)	3 (12)

*ER* emergency room, *FFP* fresh frozen plasma, *1QR* interquartile range, *PRBC* packed red blood cells and REBOA resuscitative endovascular balloon occlusion of the aorta; * *p* < 0.05 vs. trauma group

**Table 3:** Characteristics of non-trauma patients

No.	Age	Sex	SI	Glasgow- Blatchford score	Clinical Rock all score	Diagnosis	Treatment	Sheath insertion	Position (Zone)	CPA during procedure	Intervals for REBOA (miri)	REBOA-related complications	Outcome			Cause of death
													ER	24 h>	3monts>	
17	69	M	1.6	13	3	Gastric ulcer	Surgery	Success	I	No	46	None	Alive	.Alive	.Alive	-
IS	50	M	1.0	12	2	Duodenal ulcer Pseudo - aneurysm	AE (failed endoscopy)	Success	I	No	50	None	Alive	Alive	Alive	-
19	64	M	2.1	11	3	by pancreatic fistula	AE	Success	I	Yes	54	None	Alive	Alive	Alive	-
20	S3	M	2.1	19	4	Duodenal ticer	Endoscopy	Success	I	No	140	None	Alive	Alive	Alive	-
21	36	M	0.7	7	3	Gastric ulcer	Endoscopy	Success	I	No	20	None	Alive	Alive	Alive	-
22	69	M	2.S	17	3	Gastric ulcer	Endoscopy	Success	I	No	57	None	Alive	Alive	Alive	-
23	72	M	1.2	9	3	Gastric ulcer/ Cerebral infarction	AE (failed endoscopy)	Success	I	Yes	55	None	Alive	Alive	Dead	Exsanguination
24	69	M	1.7	12	3	Duodenal ulcer	AE (failed endoscopy)	Success	I	Yes	95	None	Alive	.Alive	Dead	Ischemic encephalopathy
25	78	M	O.S	14	5	Duodenal ulcer	AE (failed endoscopy)	Success	I	No	145	None	Alive	Dead	-	Exsanguination

*SI* shock index, *CPA* cardiopulm onary arrest, *REBOA* resuscitative endovascular balloon occlusion of the aorta, *ER* emergencyroom, *AE* angioembolizatoin

In Table 1, the Glasgow-Blatchford score in the Non-trauma group was mistakenly left blank. This should have read: ‘12 (11–14)’. Additionally, the abbreviations listed in the Table footnote were inconsistent with the abbreviations found in the Table itself. *APACHE* and *ICU* should not have been included in the footnote, and *FFP* (fresh frozen plasma) and *PRBC* (packed red blood cells) were omitted by mistake. A revised Table 1 is published in this erratum.

In Table 3, the ages of patients no. 17, 19 and 25 were incorrectly given in the Table. These ages should have been 64, 78 and 69 respectively. Also, the Diagnosis of patient no. 18 was incorrectly given as ‘Gastric ulcer’. This should have been ‘Duodenum ulcer’. A revised Table 3 is published in this erratum.
